# Evaluation of Germline *BMP4* Mutation as a Cause of Colorectal Cancer

**DOI:** 10.1002/humu.21376

**Published:** 2011-01

**Authors:** Steven J Lubbe, Alan M Pittman, Cornelis Matijssen, Philip Twiss, Bianca Olver, Amy Lloyd, Mobshra Qureshi, Nathan Brown, Emma Nye, Gordon Stamp, Julian Blagg, Richard S Houlston

**Affiliations:** Section of Cancer Genetics, Institute of Cancer ResearchSutton SM2 5NG, UK; Medicinal Chemistry, Cancer Research UK Centre for Cancer Therapeutics, Institute of Cancer ResearchSutton SM2 5NG, UK; Experimental Pathology Laboratory, Cancer Research UK London Research Institute44 Lincoln/s Inn Fields, London WC2A 3PX, UK; Royal Marsden Hospital, Fulham Road, London SW3 6JJ, UK; and Imperial College Faculty of Medicine, Imperial College Hammersmith CampusDu Cane Road, London W12 ONN, UK

**Keywords:** bone morphogenetic protein-4, *BMP4*, rare mutations, colorectal cancer

## Abstract

Transforming growth factor-â (TGF-â) signalling plays a key role in colorectal cancer (CRC). Bone morphogenetic protein-4 (BMP4) is a member of the TGF-â family of signal transduction molecules. To examine if germline mutation in *BMP4* causes CRC we analysed 504 genetically enriched CRC cases (by virtue of early-onset disease, family history of CRC) for mutations in the coding sequence of *BMP4*. We identified three pathogenic mutations, p.R286X (g.8330C>T), p.W325C (g.8449G>T) and p.C373S (g.8592G>C), amongst the CRC cases which were not observed in 524 healthy controls. p.R286X localizes to the N-terminal of the TGF-â1 prodomain truncating the protein prior to the active domain. p.W325C and p.C373S mutations are predicted from protein homology modelling with BMP2 to impact deleteriously on BMP4 function. Segregation of p.C373S with adenoma and hyperplastic polyp in first-degree relatives of the case suggests germline mutations may confer a juvenile polyposis-type phenotype. These findings suggest mutation of *BMP4*is a cause of CRC and the value of protein-based modelling in the elucidation of rare disease-causing variants. © 2010 Wiley-Liss, Inc.

## INTRODUCTION

Transforming growth factor-β (TGF-β; MIM# 190180) signalling plays a key role in developmental biology, cell proliferation, differentiation and migration (Xu and Pasche, 2007); and is increasingly recognized to be important in cancer biology (Tenesa and Dunlop, 2009). Furthermore, germline mutations in the genes encoding the TGF-β signalling proteins, SMAD4 (Howe, et al., 1998; MIM# 600993) and BMPR1A (Howe, et al., 2001; MIM# 601299) have been shown to cause juvenile polyposis, an autosomal dominant polyposis syndrome which confers a high risk of colorectal cancer (CRC).

Genome-wide association (GWA) studies have recently shown that common genetic variation in the gene encoding the TGF-β signalling protein *BMP4* influences CRC risk (Houlston, et al., 2008). The bone morphogenetic protein-4 (*BMP4*; MIM# 112262; NM_001202.3) gene maps to 14q22.2 and is composed of 4 exons. Multiple BMP4 transcripts are generated (Shore, et al., 1998), but only exons 3 and 4 are translated (van den Wijngaard, et al., 1996), from which a homodimer of two 408 amino acid BMP4 polypeptides is formed. Six cysteine residues form the critical protein folding motif (referred to as the cysteine knot) while a seventh cysteine is responsible for homodimerisation of BMP4 (McDonald and Hendrickson, 1993). This homodimer consisting of TGF-β1 prodomains and active domains (McDonald and Hendrickson, 1993) is proteolytically cleaved at two sites to generate the active ligand (Aono, et al., 1995). Mature BMP4 initiates TGF-β signalling by binding to type I (BMPR1A) and type II (BMPR2; MIM# 600799) serine or threonine kinase receptors, triggering intracellular SMAD-signalling (Schmierer and Hill, 2007).

It is increasingly being recognised that both common variants and coding mutations of the same gene can confer cancer susceptibility, exemplified by *CDH1* (MIM# 192090) in colorectal cancer (Houlston, et al., 2008; Richards, et al., 1999; Salahshor, et al., 2001). To examine if rare germline mutations in the coding region of *BMP4* cause CRC we analysed 504 genetically enriched CRC cases.

## METHODS

### Subjects

Five hundred and four CRC cases (298 male) were ascertained through the National Study of Colorectal Cancer (NSCCG; Penegar, et al., 2007) and the Royal Marsden Hospital NHS Trust Family History and DNA Registry (RMHNHST). All cases had histologically proven colorectal adenocarcinoma (International classification of diseases, 9th Revision [ICD9] codes 153 or 154) and none had previously been documented to have a diagnosis of a cancer syndrome known to be associated with increased CRC risk. To enhance our power to detect germline mutations, case selection was prioritized for early-age of onset (n= 182 diagnosed <55 years, family history of CRC n=250) and microsatellite stable (MSS) disease (n=210). Samples from 524 healthy individuals collected through RMHNHST (219 males; mean age at sampling 58.0 years, SD 14.0), who did not have a personal history of malignancy at time of ascertainment served as a source of controls. Both cases and controls were UK residents and had self-reported European ancestry.

The study was conducted with informed consent and ethical approval (MREC 02/0/097 and RMHNHST-CCR1552) in accordance with the declaration of Helsinki.

### Molecular analyses

Genomic DNA was salt-extracted from EDTA-venous blood samples (Miller, et al., 1988). Amplification of genomic DNA was performed with 12.5ng DNA and PCR was carried out by use of Qiagen Multiplex Kit (QIAGEN Ltd, Crawley, UK). Sequencing was performed with Big Dye version 3.1 using ABI 3730xl semi-automated sequencers (Applied Biosystems, Foster City, USA) in accordance with the manufacturer's protocol. Sequence data was analysed using Mutation Surveyor (Soft Genetics, USA) and sequence changes were annotated against GenBank contig NC_000014.8 sequence data according to the nomenclature advocated by Human Genetic Variation (HGV; den Dunnen and Antonarakis, 2000; http://www.hgvs.org/).

To assess allelic imbalance at 14q22.2-*BMP4* in the CRCs from cases carrying germline *BMP4* mutations, DNA was extracted from microdissected formalin fixed paraffin embedded (FFPE) tumors using Qiagen DNA Mini kits (QIAGEN Ltd., Crawley, UK). Loss of heterozygosity was assessed by comparing peak heights of PCR-amplified germline and tumor exon fragments encompassing mutations using ABI 3730xl semi-automated sequencers and Mutation Surveyor software.

Microsatellite instability (MSI) in CRCs was determined using BAT25 and BAT26 markers which are highly sensitive MSI markers (Boland, et al., 1998), as previously described (Penegar, et al., 2007). Samples showing novel alleles at either or both markers were assigned as MSI (corresponding to MSI-high).

To assess promoter CpG island methylation of *BMP4* (chr14:53,489,935-53,492,708 and 53,488,428-53,488,631) germline genomic DNA and tumor DNA from microdissected FFPE tumors were subjected to bisulfite conversion and were purified using the EpiTect Bisulfite kit (QIAGEN Ltd., Crawley, UK). PCR amplification of the putative BMP4 sequence was performed on eluted DNA and search for differential methylation conducted by Pyrosequencing technology (QIAGEN Ltd., Crawley, UK) using biotinylated oligonucleotide primers.

Details of all oligonucleotide primers used are shown in Supp. Table S1.

### Bioinformatic analyses

We applied two *in silico* algorithms, PolyPhen (Ramensky, et al., 2002; http://genetics.bwh.harvard.edu/pph/) and SIFT (Ng and Henikoff, 2001; http://sift.jcvi.org/), to predict the putative effect of non-synonymous coding changes in *BMP 4* on expressed protein function. Protein sequence of BMP4 (NP_001193.2) was obtained from the NCBI Human RefSeq database (Pruitt, et al., 2005; http://www.ncbi.nlm.nih.gov/refseq/). PolyPhen scores were designated probably damaging (≥2.00), possibly damaging (1.50-1.99), potentially damaging (1.25-1.49), borderline (1.00-1.24), or benign (0.00-0.99) according to the classification proposed by Xi et al. 2004. SIFT scores were classified as intolerant (0.00-0.05), potentially intolerant (0.051-0.10), borderline (0.101-0.20), or tolerant (0.201-1.00) according to the classification proposed by Ng and Henikoff, 2001 and Xi et al., 2004.

The effect of *BMP4* mutations on the stability of BMP4, as well as the ability of BMP4 to interact with other proteins, was investigated by homology modelling using Molecular operating environment v2008.10 (MOE, Chemical Computing Group Inc. Montreal, Canada). The functionally inactive prodomain could not be modelled due to lack of crystallized structures of BMP4 and/or closely homologous proteins thus the structural analysis was limited to the active domain. BMP2 (NP_001191.1) shows the greatest similarity to BMP4 with a sequence identity of 90% and a similarity of 95% for the active domain. The active domains of BMP2 and BMP4 are also of similar length. Given the high sequence identity, similarity and length between the proteins, the effect of *BMP4* mutation in the active domain can be derived directly from the known crystal structure of BMP2. This also allowed for the generation of a homology model of BMP4 based upon the BMP2 structure.

Two crystal structures of BMP2, *2goo* (Pinto, et al., 2006) and *2h64* (Weber, et al., 2007) were obtained from The Protein Databank (Berman, et al., 2000; http://www.pdb.org/pdb/home/home.do) which served as templates for generation of the homology models of BMP4. Homology models were generated using the default settings with the Amber99 forcefield and medium model refinement. An overlay of the BMP4 homology model and the BMP2 crystal structure showed that the backbones of both proteins align and that sidechains occupy the same orientation.

## RESULTS

Complete sequence data of the coding regions of *BMP4* and accompanying splice sites was obtained for all 504 CRC cases and 524 controls. One common non-synonymous single nucleotide polymorphism (nsSNP) rs17563T>C (p.V152A) was found in both cases and controls at similar frequency (minor allele frequencies 0.44 and 0.41 respectively).

**Figure 1:**
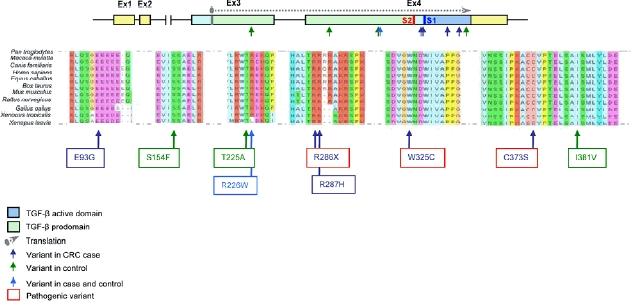
Amino acid sequence alignment of vertebrate BMP4.The upper figure shows a schematic representation of the human *BMP4* gene (NM_001202.3) with the positions of exons and their translated protein domains. Both exon 3 and exon 4 are in the translated region (grey arrow). Filled green is the TGF beta prodomain and violet is the TGF beta active domain. Positions of mutations identified are indicated for cases (dark blue arrows) and controls (green arrows). The position of R226W seen in both cases and controls is indicated by a light blue arrow. The S1 and S2 furin cleavage sites are also shown. Comparative amino acid alignment follows the order of Pan troglodytes (Chimpanzee [XP_509954]), Macaca mulatta (Rhesus monkey [XP_001084801]), Canis familiaris (Dog [XP_851628]), Homo sapiens (Human [NP_001193]), Equus caballus (Horse [NP_001157442]), Bos taurus (Cow [NP_001039342]), Mus musculus (Mouse [NP_031580.1]), Rattus norvegicus (Rat [NP_036959]), Gallus gallus (Chicken [NP_990568]), Xenopus tropicalis (Western clawed Frog [NP_001017034]), and Xenopus laevis (African clawed frog [NP_001081501]).

Five missense mutations, p.E93G (g.6788A>G), p.R226W (g.8150C>T), p.R287H (g.8334G>A), p.W325C (g.8449G>T) and p.C373S (g.8592G>C), and one truncating mutation, p.R286X (g.8330C>T), were identified in single individuals among the 504 CRC cases screened (Table 1). Four missense mutations, p.S154F (n=2; g.7935C>T), p.T225A (n=2; g.8147A>G), p.R226W and p.I381V (g.8615A>G), were identified in the 524 controls (Table 1). Comparative amino acid sequence analysis revealed complete conservation of the amino acid sequence of *BMP4* in eight species (human, chimpanzee, cow, rat, dog, chicken, frog, and zebrafish) at each of these positions (Figure 1). p.E96G is located in the functionally inactive prodomain of *BMP4* and is predicted by SIFT and PolyPhen to be benign/tolerated. p.S154F and p.R226W are both predicted to be probably damaging or to affect the protein structure, with p.T225A predicted to be benign/tolerated. However, all three mutations map to the prodomain of BMP4 and are thus unlikely to have functional consequences *a priori*.

**Table 1:** Predicted effects of non-synonymous coding changes in *BMP4* on expressed protein function

Mutation	Location in protein	Case/Control	PolyPhen score		SIFT score	
g.6788A>G; p.E93G	Prodomain	Case	0.71	Benign	0.09	Potentially intolerant
g.7935C>T; p.S154F	Prodomain	Control	1.60	Possibly damaging	0.00	Intolerant
g.8147A>G; p.T225A	Prodomain	Control	0.74	Benign	0.56	Tolerant
g.8150C>T; p.R226W	Prodomain	Case/Control	2.09	Probably damaging	0.01	Intolerant
g.8330C>T; p.R286X	Prodomain	Case	N/A		N/A	
g.8334G>A; p.R287H	Prodomain	Case	0.43	Benign	0.37	Tolerant
g.8449G>T; p.W325C	Active domain	Case	4.37	Probably damaging	0.00	Intolerant
g.8592G>C; p.C373S	Active domain	Case	3.87	Probably damaging	0.00	Intolerant
g.8615A>G; p.I381V	Active domain	Control	1.20	Benign	0.06	Potentially intolerant

g, position of mutation within genomic DNA (GenBank reference sequence NC_000014.8); N/A, Not applicable; p, position of mutation within protein (GenBank reference sequence NP_001193.2); PolyPhen, *poly*morphism *phen*otyping; SIFT, *s*orts *i*ntolerant *f*rom *t*olerant.

p.R287H was identified in a female case diagnosed with rectal CRC at age 42 (ICD9 code 154.1). The individual had concomitant colorectal adenomas at diagnosis. p.R287H is located in the prodomain adjacent to the proteolytic S1 cleavage site (-R-A-K-R-; amino acids 289-292). Cleavage of the S1 site is necessary for subsequent cleavage at the S2 site (-R-I-S-R-; amino acids 253-256) (Cui, et al., 2001). A basic residue at position at amino acid 287 is prerequisite for site recognition/cleavage by furin (Watanabe, et al., 1993). Arginine and histidine are both basic amino acids and p.R287H is therefore unlikely to have any effect on the post-translational cleavage by furin. This mutation is predicted to be benign/tolerated by PolyPhen and SIFT (Table 1).

The nonsense mutation, p.R286X, was identified in a female CRC case diagnosed with sigmoid MSS CRC (ICD9 code 153.3) at age 42 who at the time of diagnosis had no first-degree relative affected with CRC. p.R286X localizes to the N-terminal region of the TGF-β1 prodomain truncating the protein prior to the active domain (amino acids 292–408). Thus, BMP4 haploinsufficiency is likely to occur through nonsense-mediated RNA decay.

The missense mutations p.C373S and p.W325C map to the active domain of BMP4. p.C373S was identified in a female case diagnosed with sigmoid CRC (ICD9 code 153.3) at age 34. The case's father and brother who were diagnosed with colorectal adenomas at ages 67 and 36, respectively, have also been shown to carry the p.C373S mutation. In addition, one of the case's sisters, also a carrier of p.C373S, had hyperplastic polyps diagnosed at age of 35. The cysteine 373 residue is essential for the correct formation of the cysteine knot (McDonald and Hendrickson, 1993). p.W325C was identified in a male familial CRC case diagnosed with rectal disease at age 62 (ICD9 code 154.1). The patient's brother had previously died of colon cancer (ICD9 code 1539.0) at the age 60. Both p.C373S and p.W325C are likely to be detrimental to the structure of BMP4 on the basis of PolyPhen and SIFT predictions (Table 1).

Only one missense mutation, p.I381V localising to the genomic region encoding the functional TGF-β domain was identified in a healthy control. This amino acid substitution is predicted to be benign/potentially intolerant by PolyPhen and SIFT (Table 1).

**Figure 2:**
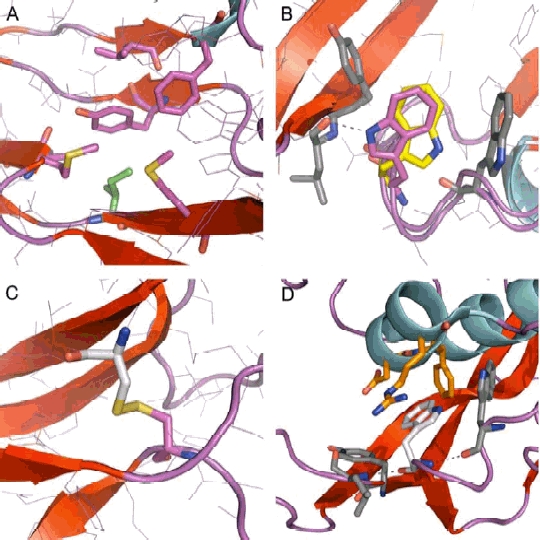
Homology modelling of BMP4 using the structure of BMP2. A: Isoleucine 381 (green) within a lipophilic pocket (purple); B: The interactions formed by tryptophan 325 in two different conformations (purple and yellow) with tryptophan 322 and tyrosine 385 (grey); C: The disulphide bridge formed by cysteine 373 (white); D: The interacting residues with tryptophan 325 (white), BMP4 (grey) and BMPR1A (orange).

To further examine the impact of p.W325C, p.C373S and p.I381V mutations we modelled the effects of these amino acid substitutions on the structure of the BMP4 protein. Isoleucine 381 interacts with other residues due to its location within a lipophilic pocket, but the p.I381V mutation changes its lipophilic properties to a limited extent thereby maintaining its interactions with the other residues. This mutation is not, therefore, predicted to impact protein stability (Figure 2A). Two crystal structures (*2goo* and *2h64*) show tryptophan 325 in two conformations. Both conformations show an edge-to-face interaction with tryptophan 322 but the tryptophan residue flips in orientation between the two conformations (Figure 2B). The p.W325C mutation reduces this edge-to-face interaction and is predicted to lower the stability of the protein. The p.C373S mutation (Figure 2C) is predicted to result in a less stable protein structure due as to the removal of a critical disulphide bond and cysteine knot motif.

Since BMP4 is known to associate with BMPR1A, protein-protein interactions were investigated between the BMP4-BMPR1A protein-complex. Tryptophan 325 of BMP4 is the only residue predicted to interact with BMPR1A as it is identical in the aligned sequence of BMP2 and BMP4. Therefore, the crystal structure of BMP2 was used as a model to analyse the effect of mutation on the protein-protein complex. Tryptophan 325 forms a π-stacking interaction with arginine 88 and forms an edge-to-face interaction with phenylalanine 85 (Figure 2D). The mutation p.W325C is thus predicted to significantly reduce these interactions and result in reduced binding of BMPR1A to BMP4. Because multiple residues are involved in binding between both proteins it is difficult to predict if this variant would result in a complete loss of the formation of the BMP4-BMPR1A complex.

Archival tumor blocks were available on CRCs from p.R286X and p.C373S. Using these we examined tumors for loss of heterozygosity indicative of BMP4 having a tumor suppressor role. In both cases no evidence of differential allelotype was seen between germline and tumor DNA (data not shown). Using paired normal and tumor DNA samples we also failed to demonstrate differential methylation of the *BMP4* promotor (data not shown).

## DISCUSSION

The BMP pathway is inactivated in a large proportion of sporadic CRC (Kodach, et al., 2008). Evidence for germline mutation in BMP4 having a role in CRC predisposition is provided by the study of inhibition of BMP4 signalling by transgenic expression of noggin in the mouse intestine. This leads to the formation of numerous ectopic crypt units, perpendicular to the crypt-villus axis, changes which typify the juvenile polyposis syndrome (Haramis, etal.,2004).

In this study we have sought to establish a direct relationship between germline mutation in *BMP4* and risk of CRC. It can be difficult to prove that a particular rare variant observed is etiologic unless those variants are strong candidates. BMP signalling inhibits intestinal stem cell self-renewal through suppression of Wnt-β-catenin signalling and disruption of this signalling pathway has strong candidacy for CRC predisposition (He, et al., 2004). Direct evidence for this assertion is provided by the fact that inactivating mutations in *SMAD4* (Howe, et al., 1998) and *BMPR1A* (Howe, et al., 2001), which, like *BMP4*, are members of the TGF-β superfamily cause the rare juvenile polyposis syndrome, which carries a very high risk of CRC. These data provide strong evidence that haploinsufficiency of BMP4 will increase CRC risk. Kryukov et al. have provided empiric evidence that about 70% of missense mutations present at population frequencies of 1% or less probably contribute to the phenotype in which they are first identified (Kryukov, et al., 2007). While we identified six mutations in both case and controls, three and five of the mutations in the cases and controls respectively map to the pro-domain of the expressed protein and are very unlikely to have any functional consequences. On the basis of detailed bioinformatics and protein homology modelling, only the three case mutations are likely to have functional consequences on the expressed protein. Sequence variations in the coding region of *BMP4*, aside from rs17563, are rare in both European (Felder, et al., 2002; Weber, et al., 2008) and Chinese populations (Suzuki, et al., 2009). On the basis of collective data on over 1,300 healthy population controls screened in our study and previously reported data (Bakrania, et al., 2008; Felder, et al., 2002; Suzuki, et al., 2009; Weber, et al., 2008) we identified three *BMP4* mutations in our CRC cohort which are predicted to functionally deleterious on expressed BMP4 (none were identified in controls) would mean that there is more than a 90% probability that at least one of these variants contributes directly to the development of CRC.

Although in part speculative the demonstration that colorectal adenomas segregated with the p.C373S mutation in the one family and the fact that the case harboring p.W325C had a first-degree relative affected with CRC suggests that *BMP4* mutations are likely to confer a moderate-high risk of CRC.

In our study we did not observe loss of the wild-type allele in CRC from mutation carriers, providing little support for a classical tumor suppressor model of carcinogenesis. Although we were unable to demonstrate differential methylation of the putative promoter region of *BMP4* we cannot fully exclude the possibility of inactivation of the wild-type allele through hypermethylation as a basis of tumor development. Accepting this caveat there exists the possibility that *BMP4* mutations may impact on risk through a dominant negative effect as has previously been shown for *SMAD4* mutations causing juvenile polyposis (Howe, et al., 1998).

Bmp signalling is a determinant of dorsal closure in *Drosophila Melanogaster* (Fernandez, et al., 2007), and in concert with Shh, growth factors and Wnt7a coordinates growth and anteroposterior, proximodistal, and dorsoventral axes morphogenesis (Basler and Struhl, 1994; Kingsley, 1994; Tickle, 1995). While we did not document any associated extra-colonic clinical feature in the cases in which we identified *BMP4* mutations, intriguingly a relationship between genetic susceptibility to clefting and cancer risk is supported in some epidemiological studies (Bille, et al., 2005; Zhu, et al., 2002). Germline mutations in *BMP4* have been suggested to be causal of cleft lip/palate (Suzuki, et al., 2009), anomalies kidney development (Weber, et al., 2008) and neural tube defects (Felder, et al., 2002). Aside from one truncating mutation (Suzuki, et al., 2009) all of the mutations on which these assertions rest are based on sequence variation predicted to have no functional consequence. Contiguous gene defects involving *BMP4* have been implicated in etiology of eye, brain, and digit anomalies (Bakrania, et al., 2008) although carrier first-degree relatives of some of the affected individuals were shown to be phenotypically unaffected.

There is increasing recognition that much of the excess familial risk of CRC is a consequence of rare disease-causing variants. The use of next generation sequencing technologies to screen CRC cases for causal variants will require enunciation of variants through bioinformatic methodologies. This study serves to illustrate the use of protein homology modelling methods for such analyses.

We have previously shown that SNPs close to the BMP antagonist, Gremlin-1 (*GREM1*; MIM# 603054) are associated with CRC risk (Jaeger, et al., 2008). Our findings provide a strong rationale for evaluating additional members of the TGF-β superfamily of genes for coding mutations. Furthermore our finding provides a strong rationale for screening other genes identified by GWA studies is likely to identify additional germline mutations, which may cause CRC.

## Figures and Tables

**Supp. Table S1:** Oligonucleotide primer pairs

**Mutation identification**	Exon 3	A-F	GGCGCTCAGGATACTCAAGA
		A-R	CTTACTTTCAGGCCGTGCAT
		B-F	AAACCCGACAAACACACAGC
		B-R	CCTAGCAAGAGTGCCGTCAT
	Exon 4	A-F	TCTCAGGGATGCTGCTGAGG
		A-R	AAGAGGGAGAGGGTGGTTGT
		B-F	ATGGCCAAAGGTGACCAG
		B-R	CCCAACCCTGAGCTATCTCTT
		C-F	CCTCAAGGACTGCCTGATCTC
		C-R	GGCTAGCCATTGAGGTGACT
		D-F	CAGGTACCACCTTTTGACTTCC
		D-R	CTGGTCAATTCTGTCAATTCCA
**Loss of heterozygosity assessment**	p.R286X	F	TCGTTACCTCAAGGGAGTGG
		R	CGCTGTGAGTGATGCTTAGG
	p.C373S	F	TGACCACCTCAACTCAACCA
		R	CATCCAGGTACAGCATGGAG
**CpG methylation assessment**	Pyrosequencing	1-F	GGATGCCACACTCACCTAGCTT
		1-R	TTTCTGGGGATGGGAGCC*
		1-S	GCCACACTCACCTAGC
		2-F	*AACCTGCTCCAGCTAGGTGTTCT
		2-R	CTACCAAGGGCTTCTCTTGTTTC
		2-S	TCAGCCCAAGACCCC
		3-F	TAGGGGCATTGGCAGGTA
		3-R	GCAGAAGCCCTTTAAAAAGCC*
		3-S	ACCAACACCTCAGGTC
		4-F	AGAAAGGAAATCCCACCATGT
		4-R	AGCAAATACTCCTTCAAGAATTT*
		4-S	GGAAATCCCACCATGT
		5-F	TAATGAGACTCCACCGCAGACA
		5-R	TGAAGCCAGAGGATTTGGGG*
		5-S	GCTCCCAGGGCTACT
		6-F	*AGGAGCTCATGGCTCTGC
		6-R	CATCAGCATTCGGTTACCAG
		6-S	CAGGTATCAAACTAGCATGG
	Sequencing	1-F	CATCGGGGAGACAAGCTAGA
		1-R	CTGGAGGGGAGGATGTGG
		2-F	AGCAGGACTGCGCTCTCTAC
		2-R	GGCAGGAGAGCAGAAACAAG
		3-F	AAAGTAAAGGATCGAACAGCTCA
		3-R	GCCTCCGTCTGCTTTCTCTA
		4-F	AATGTGGCCGAGGTTTTACA
		4-R	GGACATCCCAGTAACTGCTTG
		5-F	CTTCCAGCCCCTCGGAGT
		5-R	GAGACGCAGTCCCTTCAGTG
		6-F	ATGACGGCACTCTTGCTAGG
		6-R	CCTGGTAACCGAATGCTGAT

* Biotin label; F, forward primer; p, position of mutation within protein (GenBank reference sequence NP_001193.2); R, reverse primer; S, sequencing primer.

All oligonucleotide primer pairs *for BMP4* were designed using GenBank reference sequence NC_000014.8.
